# V_2_O_5_ encapsulated MWCNTs in 2D surface architecture: Complete solid-state bendable highly stabilized energy efficient supercapacitor device

**DOI:** 10.1038/srep43430

**Published:** 2017-03-03

**Authors:** Bidhan Pandit, Deepak P. Dubal, Pedro Gómez-Romero, Bharat B. Kale, Babasaheb R. Sankapal

**Affiliations:** 1 Nano Materials and Device Laboratory, Department of Physics, Visvesvaraya National Institute of Technology, South Ambazari Road, Nagpur 440010, Maharashtra, India; 2 Catalan Institute of Nanoscience and Nanotechnology (ICN2), CSIC and The Barcelona Institute of Science and Technology, Campus UAB, Bellaterra, 08193 Barcelona, Spain; 3 Centre for Materials for Electronics Technology (C-MET), Panchwati, Pashan Road, Pune 411 008, Maharashtra, India

## Abstract

A simple and scalable approach has been reported for V_2_O_5_ encapsulation over interconnected multi-walled carbon nanotubes (MWCNTs) network using chemical bath deposition method. Chemically synthesized V_2_O_5_/MWCNTs electrode exhibited excellent charge-discharge capability with extraordinary cycling retention of 93% over 4000 cycles in liquid-electrolyte. Electrochemical investigations have been performed to evaluate the origin of capacitive behavior from dual contribution of surface-controlled and diffusion-controlled charge components. Furthermore, a complete flexible solid-state, flexible symmetric supercapacitor (FSS-SSC) device was assembled with V_2_O_5_/MWCNTs electrodes which yield remarkable values of specific power and energy densities along with enhanced cyclic stability over liquid configuration. As a practical demonstration, the constructed device was used to lit the ‘VNIT’ acronym assembled using 21 LED’s.

One dimensional (1D) carbon nanotubes (CNTs) are well-suited for supercapacitor applications due to their great conductive nature, porous surface area, and high aspect ratio. However, improved performances could be gained by encapsulating its surface with other electroactive materials, like transition metal oxides (TMOs). The resulting hybrid electrodes can feature dual supercapacitive behavior, as electric double layer along with pseudocapacitive from TMOs. Furthermore, TMOs possess an unmatched combination of properties like unique morphology and stability at wider temperature range that brand them superior to polymers towards supercapacitor application^1^,^2^. Amongst metal oxides, ruthenium oxide (RuO_2_) got much attention in past decades because of its reversible oxidation states and great electrical conductivity. Zhang *et al*.^3^ were able to attain a high specific capacitance of 860 F g^−1^ for tubular ruthenium oxide. On the other hand, RuO_2_ is rare, expensive and most importantly toxic, which deters as supercapacitor material^4^ and hence, research on alternative TMOs electrode materials is of great importance.

Layered V_2_O_5_ has gained much interest due to its multiple valance states responsible to deliver high specific energy based on intercalation/deintercalation of electrolyte ions^5^, but its final performance differs greatly depending on the synthetic route and the phase obtained. Sol-gel derived nanoporous V_2_O_5_ attains capacitance of 214 F g^−1^ in 2 M KCl^6^ whereas the same electrolyte at the same concentration through co-precipitation method yields 349 F g^−1^ specific capacitance^7^,^8^. Instead of using just the metal oxide, nanocomposites with MWCNTs will further boost the electrochemical properties of the resulting electrodes. A series of different metal oxide/MWCNTs composites have been used as electrode. Many strategies are auctioned to trigger the supercapacitor performance of V_2_O_5_ with the tools such as synthesis method, morphology and so forth. Kim *et al*.^9^ improved specific capacitance to 150 F g^−1^ using aqueous 6 M KOH electrolyte for V_2_O_5_/carbon fiber composites (CNFCs) prepared by electrospinning. Wu *et al*.^10^ improved electrochemical properties by sol-gel synthesized porous V_2_O_5_/graphene hybrid aerogels that allowed for an enhanced specific capacitance of 486 F g^−1^, but the values obtained for V_2_O_5_ are still under those reported for RuO_2_. According to our survey, there are very few reports on the improvement of supercapacitive properties of V_2_O_5_ with the help of highly stable carbon materials or specified synthesis conditions.

In order to achieve our targeted goal of reaching the highest possible value of specific capacitance, focus has been made on the use of V_2_O_5_ flakes encapsulating 1-D MWCNTs nanonetwork in 2-D thin film form. A simple and scalable chemical route was explored to achieve high surface area V_2_O_5_ flakes on MWCNTs to form complete flexible solid-state symmetric supercapacitor (FSS-SSC) device. The following steps are involved (i) growth of V_2_O_5_ on the surface of MWCNTs through chemical route affording advanced electro-active porous cavities for electrolyte ions, (ii) such chemical route can be integrated for large area deposition by which structural porosity can be enhanced, (iii) the diffusion and relaxation time constants of the device have been evaluated to get some insight on the superior performance, specifically the great value of specific capacitance, specific energy/power with high rate of cyclic stability of electrode, (iv) fabrication of FSS-SSC device using PVA-LiClO_4_ gel electrolyte as a mediator with targeted aspect of flexibility, enhancement in stability in well defined steady manner, and (v) to demonstrate real-world applicability by lighting ‘VNIT’ acronym assembled using 21 red LEDs by means of our FSS-SSC device with dimensions 4 × 3 cm^2^.

## Outcomes and Discussion

### Kinetics of film formation

**Figure 1:**
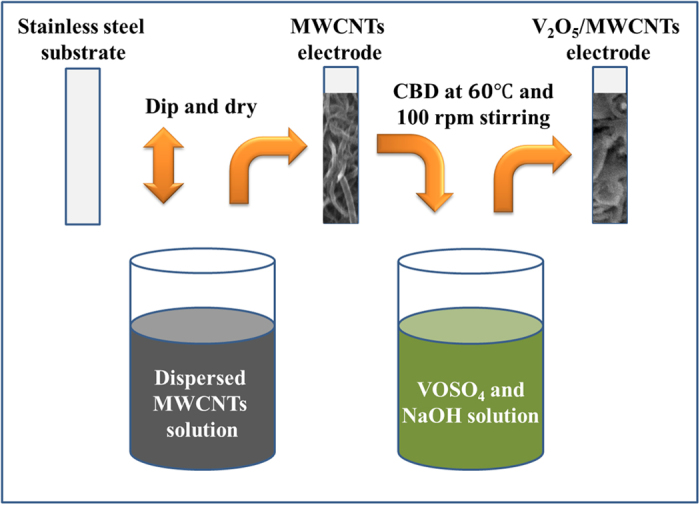
Schematic illustration for the deposition of V_2_O_5_ flakes on MWCNTs.

V_2_O_5_ flakes were grown on MWCNTs by a chemical bath deposition (CBD) method (Fig. 1). Vanadyl ion (VO^2+^) bath was prepared by dissolving VOSO_4_ into deionized water together with NaOH. At the interfacial site of MWCNTs electrode immersed in bath, VO^2+^gets oxidized to deliver V_2_O_5_ as shown in the following reaction^11^:


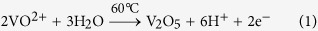


Interestingly the process consists of two stages, the first one involving the oxidation of VO^2+^ to soluble 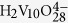:


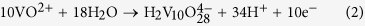


In the next stage, precipitation occurs on the electrode interface as:


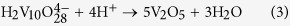


The well-dispersed precipitate present in solution subsequently condensed on stainless steel (SS) substrate as thin film.

### Structural analysis

**Figure 2:**
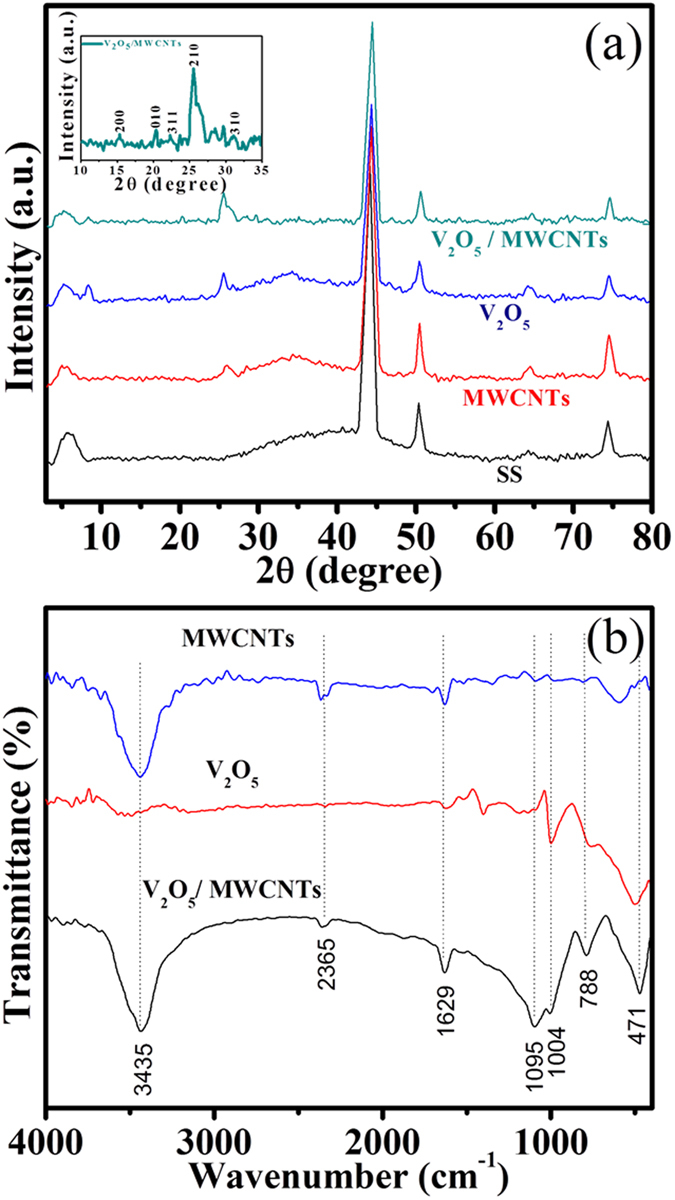
(**a**) X-ray diffraction patterns of bare stainless steel (SS), MWCNTs, V_2_O_5_ and V_2_O_5_/MWCNTs thin films, (**b**) FTIR spectra of MWCNTs, V_2_O_5_ and V_2_O_5_/MWCNTs samples.

Structural analysis of V_2_O_5_/MWCNTs on SS substrate was performed along with SS, MWCNTs and V_2_O_5_ as references (Fig. 2a). X-ray diffraction (XRD) analysis of V_2_O_5_/MWCNTs film clearly indicates an additional peak at 25.82 which resembles (002) plane of CNTs^12^. The prominent appearance of V_2_O_5_ peak at 2θ = 25.46° corresponds to (210) plane of orthorhombic V_2_O_5_ (JCPDS No. 89–2483). Moreover, the other peaks appearing at 2θ values of 15.35, 20.28, 22.74 and 30.99 degrees correspond to (200), (010), (311), (310) planes of V_2_O_5_, respectively where the other peaks belong to SS substrate. The presence of the peak at 8° in the XRD patterns of V_2_O_5_ and V_2_O_5_/MWCNTs is due to the formation of a well-ordered intercalation compound possibly resulting from the expulsion of intercalated water molecules from the nanostructure^13^,^14^,^15^.

For chemical composition and bond analysis, FTIR investigations of MWCNTs, V_2_O_5_ and V_2_O_5_/MWCNTs were performed (Fig. 2b). The triply coordinated oxygen atom between three vanadium atoms contributes to a stretching absorption^16^ peak around 471 cm^−1^. The characteristic peak at 788 cm^−1^ can be assigned to the asymmetric stretching of V–O–V bridge whereas the mode at 1004 cm^−1^ relates to terminal stretching of oxygen in V = O bond^17^. As MWCNTs were functionalized using H_2_O_2_ and forming carboxylic acid group, the stretching of C = O and O-H vibrations appeared at 1629 and 3435 cm^−1^, respectively^18^. The additional peaks at 2365 and 1095 cm^−1^ are mainly assigned to stretching modes associated to the carbon backbone^19^. In conclusion, the FTIR confirms the formation of V_2_O_5_ on MWCNTs interface with strong interaction between both components.

### Surface architecture

**Figure 3:**
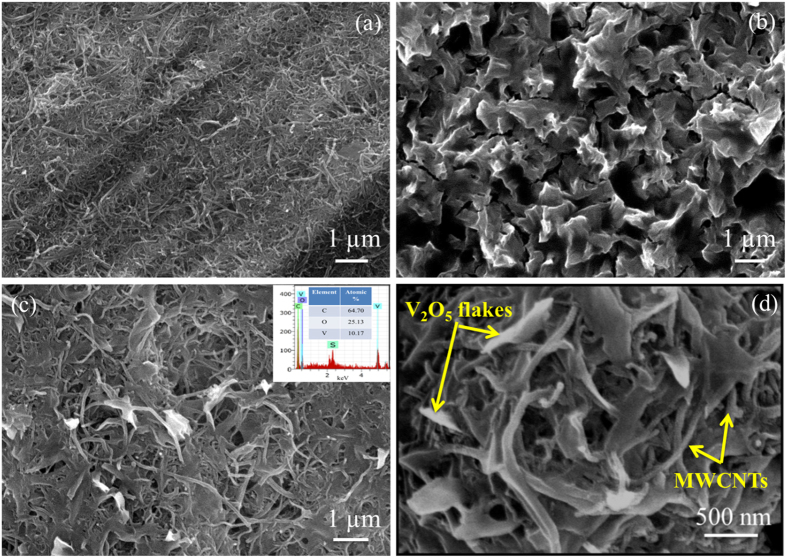
(**a–c**) FESEM images of MWCNTs, V_2_O_5_ and V_2_O_5_/MWCNTs thin film at 1 μm magnification, inset of figure (c) shows EDX spectra of V_2_O_5_/MWCNTs thin film with atomic percentage (**d**) FESEM of V_2_O_5_/MWCNTs at 500 nm.

Randomly oriented MWCNTs with well-established nanonetwork morphology can be clearly distinguished through Fig. 3a whereas Fig. 3b demonstrates unique porous intermixed flakes surface morphology of V_2_O_5_ on SS substrate. Interestingly, mirror image of similar surface architecture of V_2_O_5_ reflects on MWCNTs which is depicted in Fig. 3c,d with different magnifications. The EDX analysis (Fig. 3c**, inset**) agrees with atomic percentage values of 64.70, 25.13 and 10.17% for carbon (C), vanadium (V) and oxygen (O), respectively, which roughly confirms the presence of V_2_O_5_ with an atom percent ratio of V:O::5:2.

**Figure 4:**
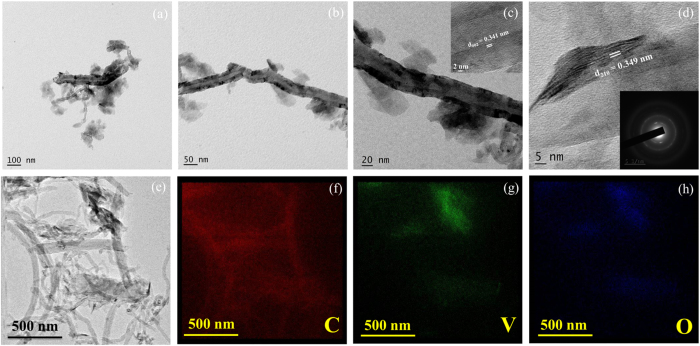
(**a–d**) HRTEM images of V_2_O_5_/MWCNTs at different magnifications, respectively, inset of figure (**c**) shows interplaner spacing of MWCNTs, and inset of figure (**d**) shows SAED pattern of V_2_O_5_/MWCNTs (**e–h**) EDS elemental mapping analysis; the red, green and blue color represents carbon (C), vanadium (V) and oxygen (O) respectively.

Important information about particle size, shape and distribution can be gathered from the TEM images. Thus Fig. 4 shows how V_2_O_5_ flakes are well anchored on 1-D MWCNTs in elongated direction resembling high wall surface in 2-D surface architecture. The V_2_O_5_/MWCNTs may provide rough surface in comparison with MWCNTs thus providing an optimal interface for rapid ion intercalation and as a consequence, superior behavior of the supercapacitor. The HRTEM images undoubtedly indicates a layer structured CNT wall with interlayer spacing of 0.341 nm (Fig. 4c), analogous to the (002) graphite plane^20^. The inter-plane spacing of 0.349 nm corresponds to (210) plane indicating orthorhombic phase of V_2_O_5_, which well supports the results of structural studies (Fig. 4d). The selected area electron diffraction (SAED) was analyzed to investigate the crystalline individualities of V_2_O_5_/MWCNTs thin film (Fig. 4d, inset). Thus, HRTEM and XRD pattern linked lattice planes have confirmed about the deposition of crystalline V_2_O_5_ on MWCNTs. The EDS elemental mapping (Fig. 4e–h) has performed during TEM investigation shows the distribution profile of carbon (C), vanadium (V) and oxygen (O); confirming a homogeneous distribution of V_2_O_5_ flakes on MWCNTs surface, whereas different contrasts reveal the different amount of material present during investigation.

### Supercapacitive electrochemical studies

**Figure 5:**
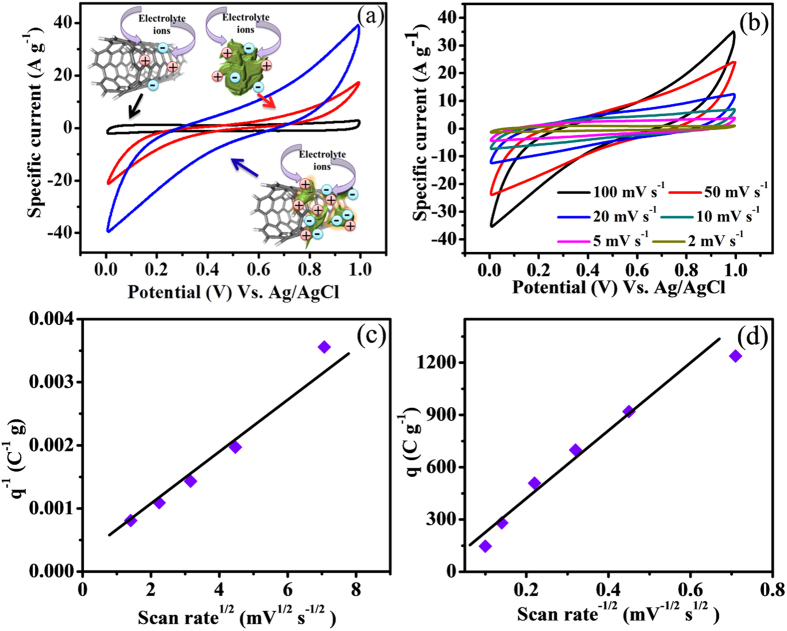
Electrochemical performances in 2 M LiClO_4_ electrolyte. (**a**) CV for MWCNTs, V_2_O_5_ and V_2_O_5_/MWCNTs samples at scan rate of 100 mV s^−1^, (**b**) CV curves of V_2_O_5_/MWCNTs at different scan rates ranging from 2 to 100 mV s^−1^, (**c**) q^−1^ vs. and v^1/2^ (**d**) q vs. v^−1/2^, plots derived from cyclic voltammograms at altered scan rate, confirming the supercapacitive characteristics.

Cyclic-voltammetric (CV) investigations of MWCNTs, V_2_O_5_ and V_2_O_5_/MWCNTs were performed in aqueous 2 M LiClO_4_ electrolyte with scan rate 100 mV s^−1^ in 0 to 1 V (Vs. Ag/AgCl) potential window at room temperature (27 °C) demonstrating a higher capacitive current for composite electrodes as compared to the individual components (Fig. 5a). The EDLC behavior of MWCNTs is evident through the rectangular shaped CV curve and the V_2_O_5_ shows reversible electrochemical nature whereas the composite exhibits the combination of both mechanisms. Intercalation/deintercalation to the active sites of electrode material with Li^+^ ions of redox species might be the basic reason assigned to the origin of the redox mechanism as per literature^16^,^21^,^22^,^23^. The electrochemical insertion of Li^+^ ions in nanostructured porous V_2_O_5_ material can be described by the following reversible redox reaction.


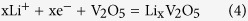


At higher scan rates, the shape of CV curves didn’t change apparently (Fig. 5b). On the other hand, an enhancement in specific current was observed with rise in scan rate indicating the excellent supercapacitive behavior of the obtained electrode material^24^,^25^.

Interestingly, supercapacitive property originates from the dual contributions as surface-controlled and diffusion-controlled charge components. During fast scan rates, diffusion of electrolyte ions is restricted to available porous cavities of electrode surface due to time constraints; resulting the fact that the charge (q) falls by rise in the scan rate (v)^26^. The outer charge (q_o_) contribution is projected by outer electrochemical surface indicated by direct contact region of electrode and electrolyte. But the inner charge (q_i_) contribution is from the sections of voids, grain boundaries, pores, crevices, and cracks etc^27^. The overall charge (q_t_) which includes the whole outer and inner active cavities assessed after the extrapolation obtained from ‘1/q’ versus ‘v^1/2^’ (Fig. 5c) assuming ‘v’ goes to zero. On the other hand, ‘q_o_’ can be calculated from ‘q’ versus ‘v^−1/2^’ (Fig. 5d) assuming ‘v’ goes to infinite^28^. Hence, one can easily determine contribution of the ‘q_i_’ after the change among ‘q_o_’ and ‘q_t_’. The evaluated results of inner (3165 C g^−1^) and outer charge (131 C g^−1^) strongly suggest the dominance of reversible redox reactions by 96% inner charge influence to specific capacitance of V_2_O_5_/MWCNTs electrode. This is because of the involvement of V_2_O_5_ electrode where almost 99% dominance of reversible redox reactions is perceived (Supplementary information S4).

**Figure 6:**
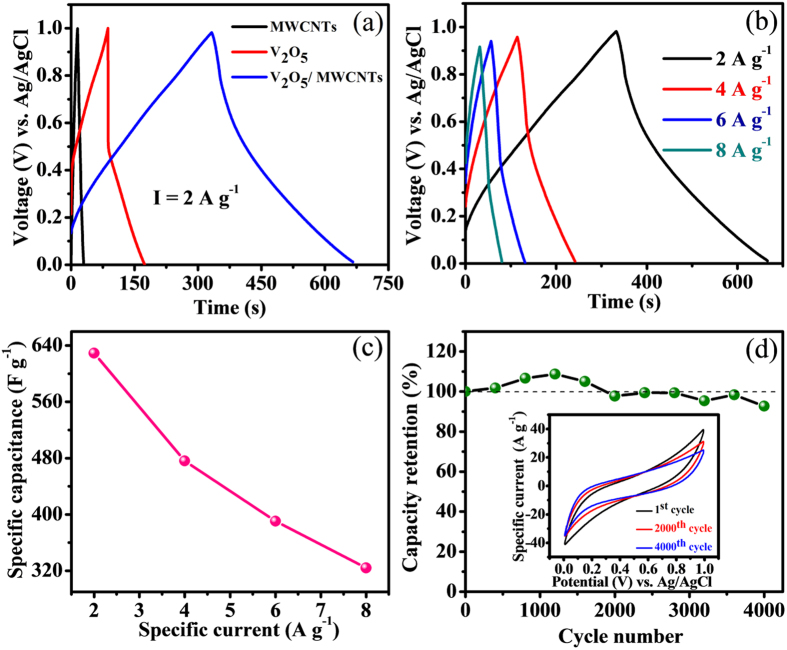
(**a**) CD curves of MWCNTs, V_2_O_5_ and V_2_O_5_/MWCNTs at specific current of 2 A g^−1^, (**b**) CD curves at different specific currents ranging from 2 to 8 A g^−1^, (**c**) specific capacitance at a function of specific current, (**d**) Cycling stability for 4000 cycles, inset shows the CV curves for different cycle numbers at 100 mV s^−1^ scan rate.

The initial three discharge-charge cycle profiles for the prepared MWCNTs, V_2_O_5_, and V_2_O_5_/MWCNTs flakes electrodes at a specific current of 2 A g^−1^ are shown in Fig. 6a. The non-faradic MWCNTs electrode provides almost triangular shaped charge-discharge profile as expected. But the non-linear shapes of V_2_O_5_ and V_2_O_5_/MWCNTs electrodes reveal three different regions consisting of (i) rapid IR drop due to internal resistance, (ii) linear section because of electrochemical double layer actions, and (iii) curved variation for reversible electrochemical activities^29^. The initial IR drop is reduced for V_2_O_5_/MWCNTs with respect to V_2_O_5_ may be due to the minimization of inner resistance in existence of MWCNTs where it provides conducting paths to the substrates. The supercapacitance values achieved at specific current of 2 A g^−1^ for MWCNTs, V_2_O_5_ and V_2_O_5_/MWCNTs electrodes are 27, 165 and 629 F g^−1^, respectively. The flakes morphology of V_2_O_5_ on MWCNTs gives more electroactive cavities for electrolyte ion diffusion to the V_2_O_5_/MWCNTs surface. So, counter ions can easily penetrate inner layer of the V_2_O_5_ where it reach at the surface and inner of the MWCNTs resulting the maximum utilization of the electrode materials. Moreover, defects and dislocations of MWCNTs can contribute additional lithium storage capacity. Charge-discharge (CD) cycles of porous V_2_O_5_/MWCNTs electrode at various specific currents (2–8 A g^−1^) are presented in Fig. 6b confirming the agreement between CD profile and CV summary in the field of supercapacitive standards.

The effectiveness of supercapacitor electrode is determined by electrochemical stability measurement by analyzing the CV profiles of V_2_O_5_/MWCNTs composite electrode at scan rate of 100 mV s^−1^ for 4000 cycles. The overlapping of CV curves without much alteration even after 4000 cycles confirms a very good cycling stability of electrode material (Fig. 6d). During the first few cycles, the CV intensities got slightly reduced, but after 250 cycles, they featured near 100% retention. The specific capacitance increases up to a maximum retention value of 109% after 1200 cycles and then reduces slowly to 93% retention at 4000 cycles which is more stable than pure V_2_O_5_ electrode (Supplementary information Figure S5). The charge incorporation including both positive and negative sweeps facilitated to rise in stability retention of electrode^30^. The excellent cycling stability confirms the strong synergy between MWCNTs and V_2_O_5_; leading to strongly enhanced electrochemical performance and mechanical stability of electrode^31^.

### Fabrication and performance evaluation of FSS-SSC device based on V_2_O_5_/MWCNTs electrodes and PVA-LiClO_4_ gel electrolyte

**Figure 7:**
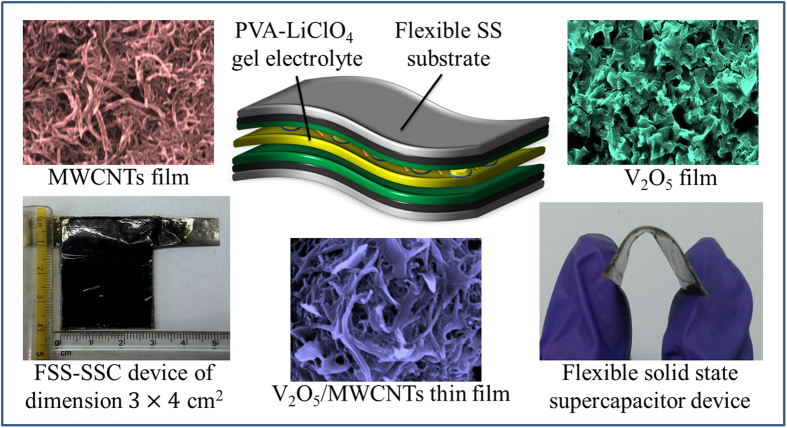
Schematic representation of the fabricated FSC-SSC device based on V_2_O_5_/MWCNTs electrode with PVA-LiClO_4_ gel as separator and electrolyte.

Considering the highly reversible electrochemical redox processes of V_2_O_5_ electrode and the fast electric double layer formation for the highly stable MWCNTs material, a FSS-SSC was fabricated using PVA-LiClO_4_ gel electrolyte (Fig. 7) to appreciate the electrochemical behaviors with steady operations. The electrochemical measurements were performed under ambient conditions.

The polymer gel fulfilled a dual behavior as electrolyte as well as separator and was prepared to manufacture symmetric device as follows: 3 g of polyvinyl alcohol (PVA) was well dispersed in 30 ml double distilled water (DDW) with constant stirring of 25 min resulting in a clear solution at 70 °C, followed by addition of 10 ml of 2 M LiClO_4_ creating a consistent gluey form^32^,^33^. A layer of this gel was used to sandwich two electrodes and a pressure of 1 ton on flat electrode surface was exposed overnight to ensure good adhesion and complete solvent evaporation which leads to the formation of flexible solid-state supercapacitor (FSS-SSC) device.

**Figure 8:**
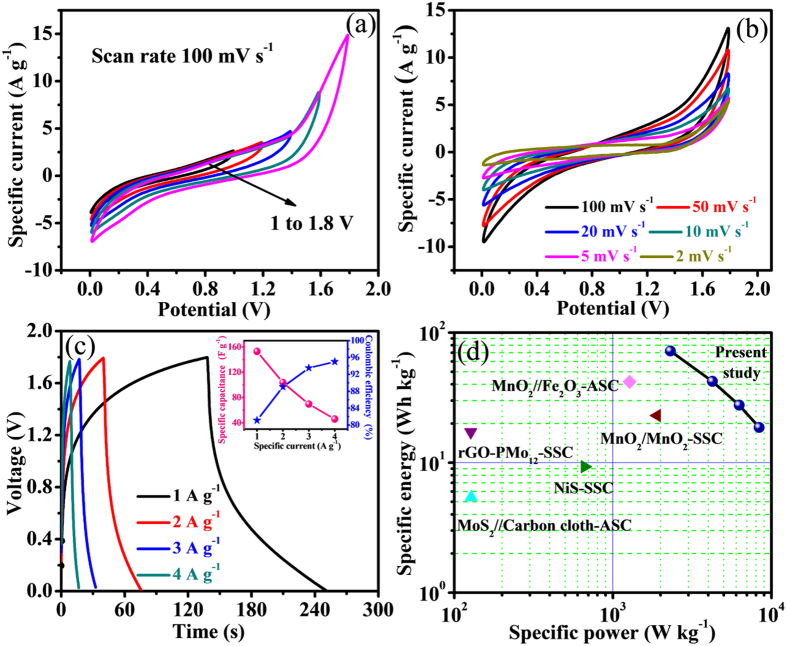
Electrochemical performance of FSS-SSC device with PVA-LiClO_4_ gel electrolyte. (**a**) CV curves for different potential windows ranging from 1 to 1.8 V at scan rate 100 mV s^−1^, (**b**) CV curves at different scan rates ranging from 2 to 100 mV s^−1^ with voltage window 1.8 V, (**c**) CD curves at different specific currents ranging from 1 to 4 A g^−1^, inset shows Specific capacitance and Coulombic efficiency as a function of specific current, (**d**) Ragone plot compared with earlier stated papers.

The fabricated device demonstrates perfect supercapacitive activity through an extended potential window from 0 to 1.8 V (Fig. 8a). Figure 8b shows CVs of FSS-SSC device broadening between 2 to 100 mV s^−1^ within operating potential frame of 1.8 V. The symmetric increment of specific current with scan rate strongly suggests the excellent supercapacitive behavior of this device. The CV shapes maintain its unique character and reversibility even at lower scan rate indicating good supercapacitive actions.

CD profiles at different constant specific currents were examined to study the rate capability and are depicted in Fig. 8c where reversible redox mechanism is the reason for the asymmetric CD curves. A superior specific capacitance of 160 F g^−1^ was reached at 1 A g^−1^ specific current with the voltage window of 1.8 V. The capacitance value of V_2_O_5_/MWCNTs symmetric cell decreases at upper specific currents according to diffusion limited charge transfer process. A Coulombic efficiency of 81% was obtained at 1 A g^−1^ and increased to 95% (at 4 A g^−1^) for the solid state device (Fig. 8c**, inset**). The excellent Coulombic efficiency originated from the intercalation/deintercalation of electrolyte ions into the orthorhombic sites of V_2_O_5_ supported by MWCNTs. The maximum specific energy obtained for FSS-SSC device was 72 Wh kg^−1^ consuming specific power of 2.3 kW kg^−1^ at 1 A g^−1^ (Fig. 8d). After increasing the specific current to 4 A g^−1^, the specific energy remains 18.66 Wh kg^−1^ with a high specific power of 8.4 kW kg^−1^ (Table 1). The device demonstrates comparatively greater specific energy than conventional ultracapacitors and ordinary capacitors, delivering specific power significantly larger than the common fuel cell and battery. The device has relatively high energy density than previously reported carbon based flexible all-solid state symmetric supercapacitor devices such as NiS (9.3 Wh kg^−1^)^34^, MnO_2_ (23 Wh kg^−1^)^35^, MnO_2_//Fe_2_O_3_ (41.8 Wh kg^−1^)^36^, rGO-PMo_12_ (17.20 Wh kg^−1^)^37^, and MoS_2_/carbon cloth (5.42 Wh kg^−1^)^38^.

**Figure 9:**
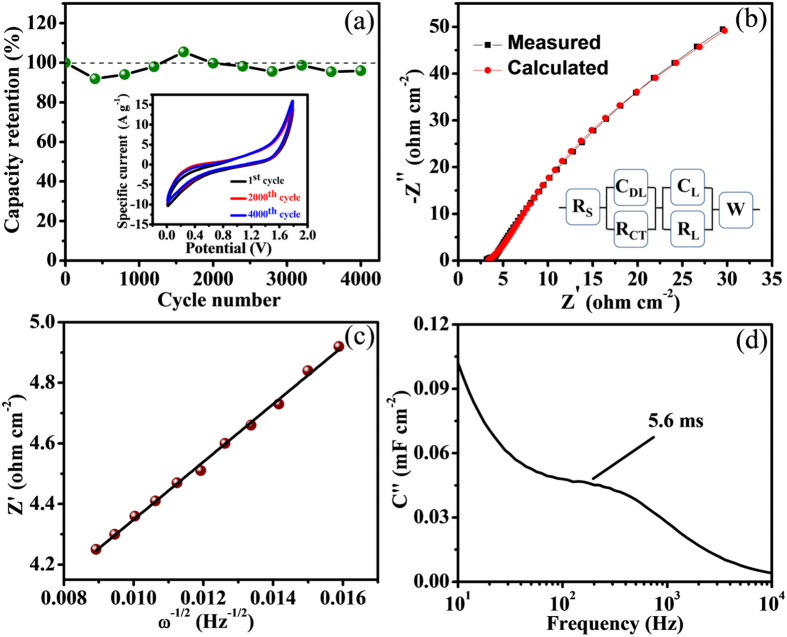
(**a**) Cycling stability for 4000 cycles at 100 mV s^−1^ scan rate, inset shows CV curves for different cycle numbers at 100 mV s^−1^ scan rate (**b**) Nyquist plot of impedance from 10 mHz to 100 kHz, inset shows corresponding equivalent circuit, (**c**) Z′ vs. ω^−1/2^ plot to evaluate diffusion coefficient, (**d**) imaginary capacitance vs. frequency plot to compute relaxation time constant.

The practical implementation of FSS-SSC device strongly depends upon long time cycling stability. The cycling constancy of fabricated FSS-SSC device was verified up to 4000 cycles at steady scan rate of 100 mV s^−1^ (Fig. 9a). There was a primary drop of specific capacitance at the beginning of few cycle, but increases to 105% retention after 1200 cycles. After 4000 cycles, it shows an excellent retention of 96% which is higher than the achieved through liquid configuration and strongly favors the commercial use of FSS-SSC device. The stability performance is comparable to previously reported devices^34^,^35^,^36^,^38^ (Table 2).

Impedance Nyquist curve of device consisting the imaginary impedance component as a function of the real part in frequency array from 100 kHz to 10 mHz was measured (Fig. 9b) which reveals the intrinsic electrochemical and kinetics mechanism of the device. The intercept in X-axis bounces the total internal resistance (R_S_) whereas the diameter of trivial arc is signified as charge transfer resistance (R_CT_). The plot demonstrates very small R_S_ and R_CT_ values as 3.2 Ω cm^−2^ and 0.84 Ω cm^−2^, respectively. The small of R_CT_ for V_2_O_5_/MWCNTs electrode greatly boosts the electrochemical behavior of manufactured device (Supplementary information S6). The lower value of R_CT_ and a transition to linearity at low frequency region strongly facilitate faster electron transport during charging and discharging^39^. The equivalent circuit is a resembled to Randles circuit which yields the R_S_ and the parallel connection of R_CT_ and C_DL_ representing the small arc in high frequency section where C_DL_ originates from double layer capacitance^40^. The straight spectra parallel to the imaginary axis acts as perfect polarized capacitive behavior which is denoted by mass capacitance (C_L_), connected parallel to R_L_ i.e. leakage resistance signifying inclined nature and Warburg element (W) represents the switch from high to low frequency region^41^.

Coating of V_2_O_5_ on MWCNTs offers more porous surface area including short diffusion path; resulting high ion diffusion coefficient and superior supercapacitive properties^42^,^43^. But the Li^+^ ion diffusion of solid-state device is restricted due to the use of solid-state electrolyte; resulting in poor electrochemical properties. Evaluation of the lithium ion diffusion coefficient (D) of the device is quite important and concluded by using Nyquist plot through the relation^44^


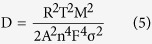


where, ‘R’, ‘T’ and ‘F’ indicates the gas constant, room temperature, and Faraday constant individually. Compound molar volume, and the area of the electrode are symbolized by ‘M’ and ‘A’, respectively. Further, the value of ‘n’ is decided by number of electrons for every molecule appearing in reaction whereas ‘*σ*’ justifies the slope of plot between Z′ vs. ω^−1/2^ (Fig. 9c), correspondingly where used standard values are R = 8.314 J mol^−1^ K^−1^, T = 298 K, A = 10^–4^ m^2^, n = 2, F = 96500 C mol^−1^, σ = 343 and M = 54.13 × 10^−6^ m^3^ mol^−1^. Diffusion coefficient of 5.5 × 12^−17^ cm^2^ s^−1^ was attained which facilitates the high rate performance during the charge-discharge capacities.

The boundary between resistive and capacitive behavior is clearly defined by the relaxation time constant (τ_0_) parameter. The low τ_0_ corresponds to high power delivery which is in favor of main supercapacive feature. The relaxation time constant (τ_0_) was calculated from 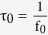 using imaginary capacitance *C*″ vs. frequency plot^45^ where


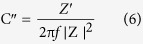


and *f*_0_ represent characteristic frequency (Supplementary information S7).

The relaxation time constant was estimated to be 5.6 ms (Fig. 9d) which undoubtedly promotes the quick ions transport rate to the interior pores of electrode material; resulting the upgraded device efficiency towards application index. The moderate values of diffusion coefficient and relaxation time constant suggest the efficient nature of FSS-SSC device.

**Figure 10:**
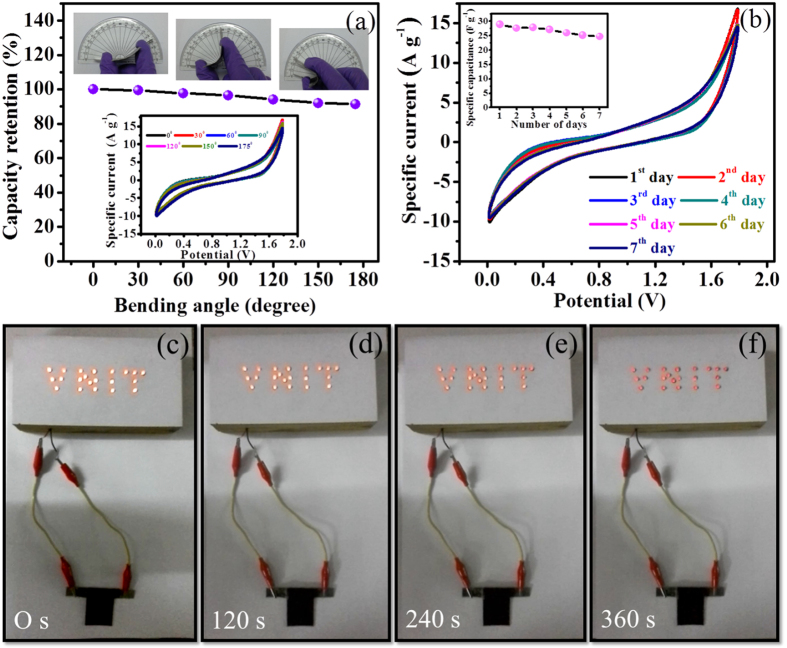
(**a**) Variation of capacity retention at different bending angles, inset shows CV curves with different bending angles at scan rate of 100 mV s^−1^, (**b**) Consistency test of fabricated FSS-SSC device for 1 week at scan rate of 100 mV s^−1^, inset shows specific capacitance changes with corresponding days (**c–f**) Actual demonstration of FSS-SSC device discharging through VNIT panel consisting 21 red LEDs in parallel arrangement at 0 s, 120 s, 240 s, and 360 s.

In the field of portable and compact electronics, mechanical flexibility is a key parameter for the formed device. For different bending angles, CV curves were measured for the same voltage window and depicted in Fig. 10a. The unperturbed shapes of CVs even at 175° bend, suggests the superb adhesion of active electrode material. The device shows 90% capacitive retention even at 175° bending angle; suggesting excellent feature of boundary concerning active electrode material and gel electrolyte. Furthermore, the outdoor stability of FSS-SSC device was measured daily for continuous one week at scan rate of 100 mV s^−1^ and we got unperturbed CV curves (Fig. 10b) yields the superior stability of the device. Such a high electrochemical parameters motivated us to demonstrate further its commercial applicability and hence, the device was charged at 1.8 V for 30 s and discharged through a panel anchoring the acronym ‘VNIT’ (Visvesvaraya National Institute of Technology) assembled using 21 red LEDs arranged in parallel combination (Supplementary video). The device surprisingly has lightened up the LED panel for 360 s (Fig. 10c–f). This lucrative FSS-SSC device can be envisaged as a superior power and energy contender in the arena of energy storage devices and portable electronics.

## Conclusions

A simple chemical bath deposition method was used to synthesize V_2_O_5_/MWCNTs electrodes which were successfully integrated into a flexible solid-state supercapacitor device. The flexible solid-state device exhibits superior cycling stability of 96% charge retention after 4000 cycles which is higher than that of the corresponding liquid electrolyte configuration. Furthermore, solid-state device features a wide potential window of 1.8 V and specific energy of 72 Wh kg^−1^ at specific power of 2.3 kW kg^−1^. High mechanical flexibility up to 175° along with the practical demonstration of lighting up ‘VNIT’ acronym assembled using 21 red LEDs further validate V_2_O_5_/MWCNTs as a new promising and emerging energy storage electrode for supercapacitor application.

## Experimental

### Encapsulation of V_2_O_5_ flakes over MWCNTs

MWCNTs in 2-d architecture have been synthesized adopting our previous report^46^. In brief, 95% pure MWCNTs with 15–20 nm outward diameter and 5–15 μm as length were procured from Nano Amor (Housten, TX). Reflux process of MWCNTs was performed using H_2_O_2_ at 60 °C for 48 hour followed by removal of amorphous carbon derivatives and anchoring of oxygenated functional groups. The obtained product was rinsed repeatedly using double distilled water (DDW) for several times and dried at 60 °C for 12 h. To obtain stable and prominent dispersion, sonication of 0.125 g of MWCNTs was performed with surfactant TritonX-100 and 25 ml double distilled water (1:100,Tx-100:water) for 1 h. Mirror cleaned stainless steel (SS, grade 305) substrate was dipped in to the MWCNTs solution for 20 s to adsorb onto SS substrate and dehydrated through IR source for solvent evaporation. Repetition of process for 20 times retains optimum coating of MWCNTs on SS and to form appropriate nucleation sites with high surface area for deposition of V_2_O_5_.

V_2_O_5_ flakes encapsulation on MWCNTs has been performed as follows: 0.4 ml of 1 M NaOH was supplemented drop by drop in 0.1 M VOSO_4_ solution to get a homogeneous solution with blue color. This solution was kept at 60 °C with 100 rpm constant stirring in which MWCNTs coated SS substrate was dipped vertically for 3 h where heterogeneous reaction resulted in to the formation of green colored V_2_O_5_. The obtained substrate was rinsed with DDW followed by subsequent drying at room temperature (27 °C). The similar process was repeated to get V_2_O_5_ thin film when SS substrate was used instead of MWCNTs coated SS substrate (Supplementary information S1).

### Characterizations

X-ray diffraction (XRD) study was acquired using Bruker AXS D8 Advance diffractometer with Cuk_α_ source (wavelength 0.15 nm) with diffraction angle (2θ) between 4 to 80°. Fourier transform infrared spectroscopy (FTIR) was performed using Thermo Nicolet, Avatar 370 in the wavenumber between 400–4000 cm^−1^. Scanning electron microscopy (SEM) coupled with energy dispersive X-Ray (EDX) spectroscopy was operated to investigate surface morphology and composition using JEOL Model JSM - 6390LV. High resolution transmission electron microscopy (HRTEM) along with EDS elemental mapping was implemented via model JEOL 2100 with LaB_6_ source. Furthermore, potentiostat (Princeton Applied Research, USA PARSTAT-4000) was engaged to evaluate electrochemical properties like cyclic-voltammetry(CV), galvanostatic charge-discharge (CD) appraisal and electrochemical impedance spectroscopy (EIS) measurements. Different electrolytes were used for electrolyte optimization and 2 M LiClO_4_ was concluded as optimized electrolyte (Supplementary information S2). Three electrode arrangement in 2 M LiClO_4_ electrolyte was adopted to measure all electrochemical behaviors in which V_2_O_5_/MWCNTs electrode acted as working electrode (WE), Ag/AgCl and platinum wire as reference electrode (RE) and counter electrode (CE) exclusively. Working electrode having dimension of 1 cm^2^ was dipped in the LiClO_4_ electrolyte solution to investigate all electrochemical properties (Supplementary information S3). For device testing, CE and RF are connected and treated as one terminal and WE as another terminal.

## Additional Information

**How to cite this article:** Pandit, B. *et al*. V_2_O_5_ encapsulated MWCNTs in 2D surface architecture: Complete solid-state bendable highly stabilized energy efficient supercapacitor device. *Sci. Rep.* **7**, 43430; doi: 10.1038/srep43430 (2017).

**Publisher's note:** Springer Nature remains neutral with regard to jurisdictional claims in published maps and institutional affiliations.

## Supplementary Material

Supplementary Video

Supplementary Information

## Figures and Tables

**Table 1:** Various parameters of the FSS-SSC device: charge time, discharge time, specific capacitance, Coulombic efficiency, specific energy, and specific power at different specific currents from charge/discharge measurements.

Specific current (A g^−1^)	Charge time (s)	Discharge time (s)	Specific capacitance (F g^−1^)	Coulombic efficiency (%)	Specific energy (Wh kg^−1^)	Specific power (kW kg^−1^)
1	138.38	112.25	160.15	81.11	72.07	2.3
2	40.07	35.68	93.52	89.04	42.08	4.2
3	16.98	15.88	61.46	93.52	27.66	6.3
4	8.44	8.02	41.46	95.02	18.66	8.4

**Table 2:** Summary of the performance of FSS-SSC device with several reported solid-state devices.

Electrode materials	Cell remarks	Specific energy (Wh kg^−1^)	Specific power (kW kg^−1^)	Cyclic stability		Ref.
				Retention (%)	Cycles	
NiS	Symmetric	9.3	0.67	90	1500	34
MnO_2_	Symmetric	23	1.9	92	2200	35
MnO_2_//Fe_2_O_3_	Asymmetric	41.8	1.3	91	3000	36
rGO-PMo_12_	Symmetric	17.20	0.13	89–95	5000	37
MoS_2_/Carbon cloth	Symmetric	5.42	0.13	96.5	5000	38
V_2_O_5_/MWCNTs	Symmetric	72.07	2.3	96	4000	Present work
