# Supplementary material for: V2O5 encapsulated MWCNTs in 2D surface architecture: Complete solid-state bendable highly stabilized energy efficient supercapacitor device
Source: Sci Rep. 2017 Mar 3;7:43430. doi: 10.1038/srep43430 (PMC5335550; doi:10.1038/srep43430)
Supplement: Supplementary Information [file srep43430-s2.pdf]

Supplementary information for:

V_2O_5 encapsulated MWCNTs in 2D surface architecture: Complete solid-state bendable highly stabilized energy efficient supercapacitor device

Bidhan Pandit[1], Deepak P. Dubal[2], Pedro Gómez-Romero[2]*, Bharat B. Kale[3], Babasaheb R. Sankapal[1]**

[1]Nano Materials and Device Laboratory, Department of Applied Physics, Visvesvaraya National Institute of Technology, South Ambazari Road, Nagpur 440010, Maharashtra, India

[2]Catalan Institute of Nanoscience and Nanotechnology (ICN2), CSIC and The Barcelona Institute of Science and Technology, Campus UAB, Bellaterra, 08193 Barcelona, Spain

[3]Centre for Materials for Electronics Technology (C-MET), Panchwati, Pashan Road, Pune 411 008, Maharashtra, India

CORRESPONDING AUTHOR FOOTNOTE

Prof. Babasaheb R. Sankapal and Prof. Pedro Gomez-Romero

E-mail: brsankapal@phy.vnit.ac.in; brsankapal@gmail.com (B. Sankapal),

Tel.: +91 (712) 2801170; Fax No. : +91 (712) 2223230

E-mail: pedro.gomez@icn2.cat (P. Gomez-Romero)

Tel.: +349373609/+34937373608; Fax No: + 34936917640

S1. Synthesis of V_2O_5 on Stainless steel substrate

Synthesis of V_2O_5 flakes on stainless steel (SS) has been performed as follows: 0.4 ml of 1 M NaOH was supplemented drop by drop in 0.1 M $VOSO_4$ solution to get a homogeneous solution with prior color blue. This solution was kept at 60° C with constant stirring of 100 rpm in which SS substrates (dimensions: 1×5 cm^2) were dipped vertically for 3 h where heterogeneous reaction resulted in to the formation of green colored V_2O_5. The obtained substrates were rinsed with DDW and then dried in air.

S2. Optimization of electrolyte and its concentration

In search of optimum electrolyte, we tested the V_2O_5 electrode in different electrolytes (Na_2SO_4, Na_2SO_3, NaOH, KOH, KCl and $LiClO_4$) at constant concentration of 0.5 M at a fixed scan rate of 100 mV s^{-1}. The electrode offered maximum specific capacitance of 19 F g^{-1} with respect to others. Now concentration of $LiClO_4$ was varied from 0.1 M to 2.5 M. The V_2O_5 electrode gave maximum specific capacitance of 42 F g^{-1} at a concentration of 2 M. Hence, all the electrochemical measurements of V_2O_5, MWCNTs and V_2O_5/MWCNTs electrodes were performed at 2 M $LiClO_4$ electrolyte to get optimum electrochemical results.

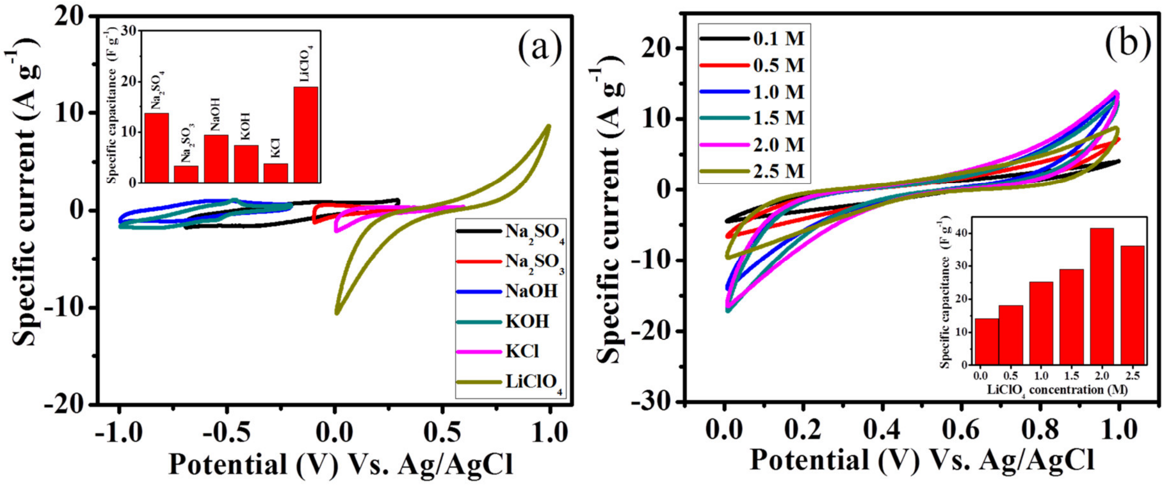

Figure S2 (a) electrolyte variation, and (b) concentration variation for optimum electrochemical behavior

S3. Electrochemical characterizations

Specific capacitance (C_s) from galvanostatic charge-discharge[1] was calculated by using the relation

$$C_s = \frac{I \int V dt}{m\left(V_f - V_i \right)^2} \qquad (1)$$

where, specific capacitance is in F g^{-1}, 'm' signifies mass (g) deposited on SS substrate, '$\left(V_f - V_i \right)$' is an functional potential frame, 'I' implies current intensity and '$\int V dt$' symbolizes the area under the experimental charge-discharge curve of the V_2O_5/MWCNTs electrode for unit area (1 cm^2) dipped in 2 M $LiClO_4$ electrolyte.

Depending upon discharging time t_d(s) and charging time t_c(s), Coulombic efficiency (η) of FSS-SSC can be assessed by

$$n = \frac{t_d}{t_c} \times 100 \qquad (2)$$

Further specific energy (E) in W h kg^{-1} associated with specific power (P) in W kg^{-1} of FSS-SSC were evaluated from the charge–discharge dimensions by using the following expressions,

$$E = \frac{1}{2}\left[\frac{C_s\left(V_f - V_i \right)^2}{3.6}\right] \qquad (3)$$

$$P = \frac{3600 \times E}{\Delta t} \qquad (4)$$

Here, 'Δt' entails discharge time (t).

S4. Inner/outer charge contribution of V_2O_5 electrode

The inner and outer charge contribution for V_2O_5/MWCNTs has already calculated which illustrates about 96% dominance of reversible redox reactions. Using similar way, we have calculated here the inner and outer charge of V_2O_5 which strongly says about 99% dominance of reversible redox reactions. As V_2O_5 flakes are encapsulated towards the electric double layer categorized MWCNTs, the slight minimization (99 to 96%) has been occurred in the field of dominancy for V_2O_5/MWCNTs over V_2O_5 electrode.

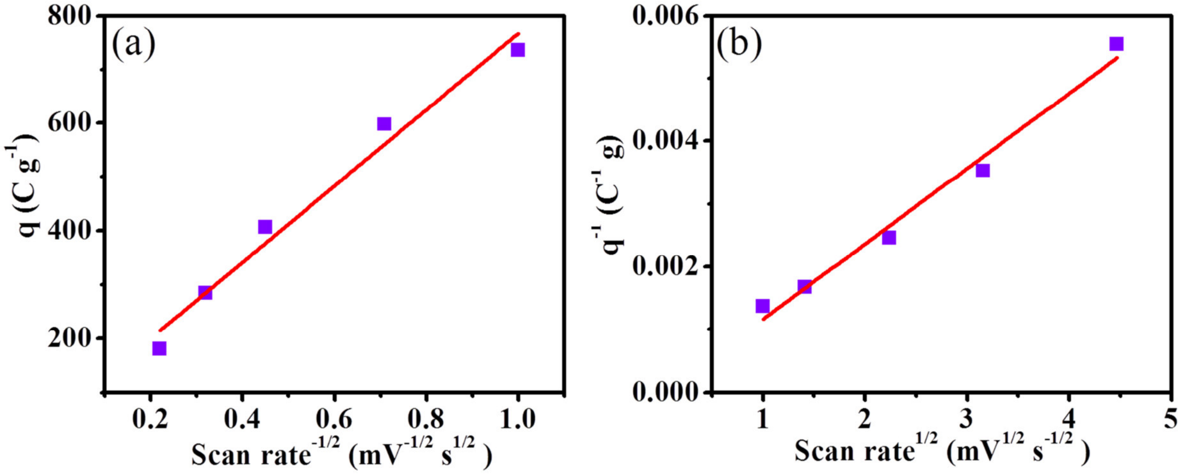

Figure S4 (c) q vs. $v^{-1/2}$ and (d) q^{-1} vs. $v^{1/2}$ plots derived from cyclic voltammograms at altered scan rate, confirming the supercapacitive characteristics

S5. Stability behavior of V_2O_5 electrode

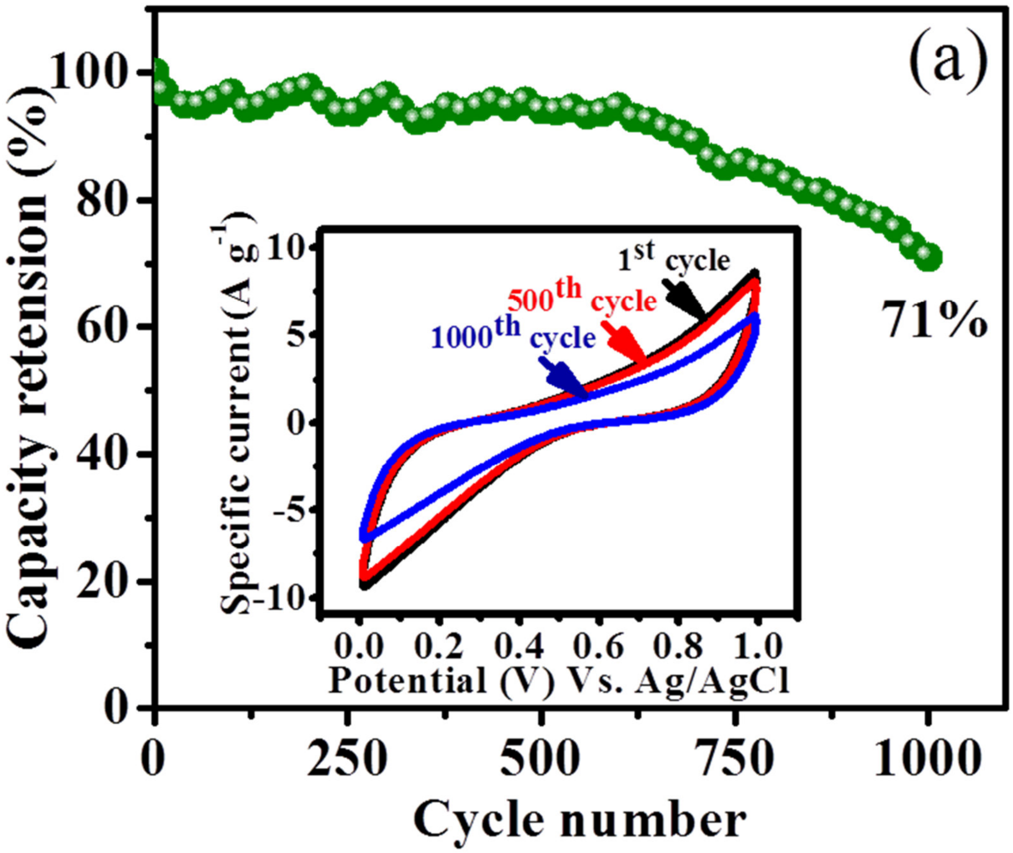

Figure S5 Cycling stability V_2O_5 electrode for 1000 cycles at 20 mV s^{-1} scan rate, inset shows the CVs for 1st, 500th and 1000th cycles

S6. EIS of V_2O_5 and V_2O_5/MWCNTs

The semi-circle indicative charge transfer resistance (R_{CT}) for V_2O_5 and V_2O_5/MWCNTs electrodes are 1.42 and 0.53 Ω cm^{-2}, respectively. The minimum value of R_{CT} for V_2O_5/MWCNTs is either due to use of MWCNTs as conducting pathway or due to the large surface area which facilitates fast intercalation/extraction of the electrolyte ions into the electrode and greatly increases the electrochemical behavior of concerned electrode material[38].

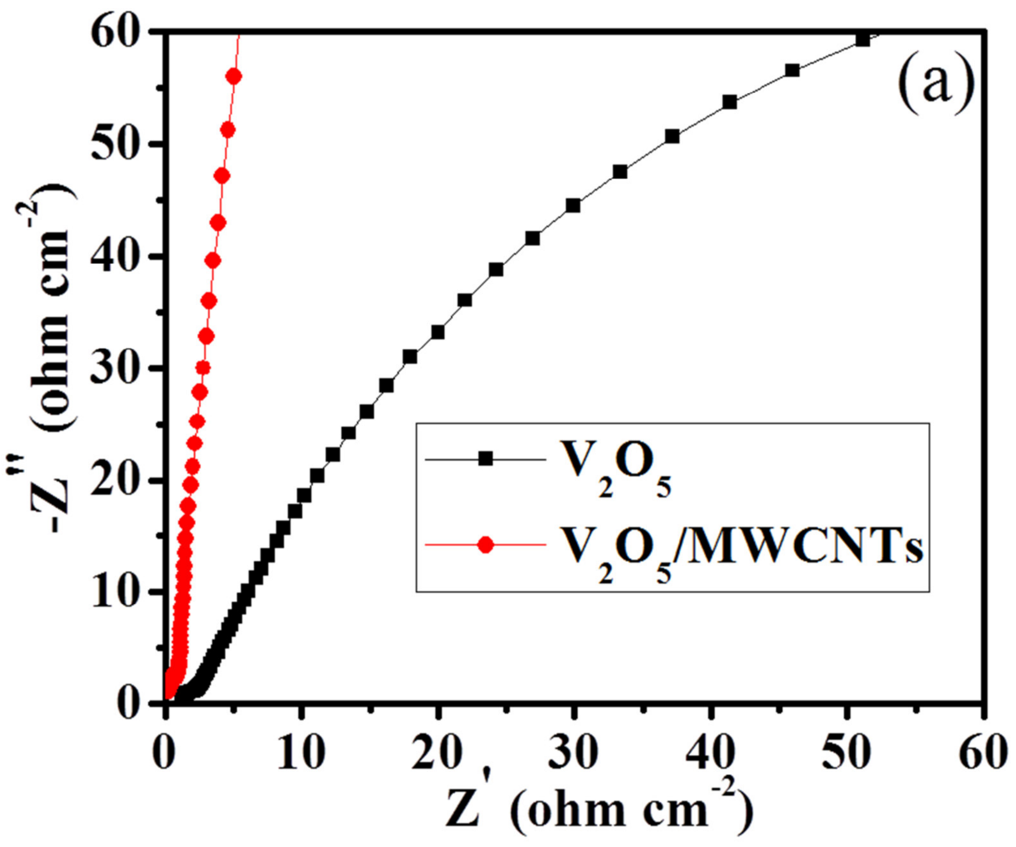

Figure S6 Nyquist plot of V_2O_5 and V_2O_5/MWCNTs for frequency ranging from 100 mHz to 100 kHz

S7. Imaginary capacitance estimation to measure relaxation time constant

We calculated $C''(\omega)$ by using following steps[2]

The impedance $Z(\omega)$ of supercapacitive component relates

$$Z(\omega) = \frac{1}{j\omega C(\omega)} \qquad (5)$$

Now in complex form, $Z(\omega) = Z'(\omega) + jZ''(\omega)$ (6)

Combining these two, we have,

$$C(\omega) = \frac{1}{j\omega\{Z'(\omega) + jZ''(\omega)\}} = \frac{-\{Z''(\omega) + jZ'(\omega)\}}{\omega \mid Z(\omega) \mid^2} \qquad (7)$$

In equivalence with, $C(\omega) = C'(\omega) + jC''(\omega)$ (8)

Easily it is derived that

$$C''(\omega) = \frac{Z'(\omega)}{\omega \mid Z(\omega) \mid^2} = \frac{Z'(\omega)}{2\pi f \mid Z(\omega) \mid^2} \qquad (9)$$

References

1 Shinde, S. K., Dubal, D. P., Ghodake, G. S., Kim, D. Y. & Fulari, V. J. Morphological tuning of CuO nanostructures by simple preparative parameters in SILAR method and their consequent effect on supercapacitors. *Nano-Struct. Nano-Objects* **6**, 5-13 (2016).

2 Portet, C., Taberna, P. L., Simon, P. & Flahaut, E. Influence of carbon nanotubes addition on carbon–carbon supercapacitor performances in organic electrolyte. *J. Power Sources* **139**, 371-378 (2005).
